# Supplementary material for: Development and preliminary testing of Health Equity Across the AI Lifecycle (HEAAL): A framework for healthcare delivery organizations to mitigate the risk of AI solutions worsening health inequities
Source: PLOS Digit Health. 2024 May 9;3(5):e0000390. doi: 10.1371/journal.pdig.0000390 (PMC11081364; doi:10.1371/journal.pdig.0000390)
Supplement: S1 Appendix — (PDF) [file pdig.0000390.s001.pdf]

S1 Appendix. Overview of Health Equity Across the AI Lifecycle (HEAAL). For evaluating an existing AI solution, follow procedures written in red and black text. For evaluating a new AI solution, follow procedures written in blue and black text.

Adoption stage	Decision point	Assessment domains	Procedures
Problem identification and procurement	1. Identify and prioritize a problem	Fitness for purpose	a. Ensure that problems are prioritized and funded equally across all patient subgroups.<br>b. Determine whether there are patient populations for whom a solution to the prioritized problem should not be used, should be used differently, or whose experience with the system should be closely monitored.
	2. Define AI product scope and intended use	Reliability and validity, Fairness	a. List alternative solutions for the problem, including non-technical interventions and other non-AI technical interventions.<br>b. Define an ideal label for model development.<br>c. Seek an approval from an institutional review board, ethical review board, or research ethics board to access and use local healthcare retrospective data.<br>d. Assess health inequities present in the local healthcare retrospective data and identify disadvantaged patient subgroups within the context of the prioritized problem.<br>e. Examine whether a local healthcare retrospective data set is representative of demographic representation of local non-healthcare data.<br>f. Assess health inequities present in the model training data and identify disadvantaged patient subgroups within the context of the prioritized problem.<br>g. Examine whether the model training data is representative of the demographics present within the local healthcare retrospective data.<br>h. Analyze label choice bias across disadvantaged and advantaged patient subgroups.<br>i. Ensure that the model features are relevant to its actual label and capture the same meanings across disadvantaged and advantaged patient subgroups.<br>j. Identify potential hidden stratification that masks unequal model performance between disadvantaged and advantaged patient subgroups.<br>k. Gather model performance data and compare it between disadvantaged and advantaged patient subgroups.<br>l. Determine which SDOH and demographic data are appropriate to be included in the model to minimize potential risk of worsening health inequities.<br>m. Determine which potential solution best solves the problem for disadvantaged patient subgroups.
Development and adaptation	3. Develop success	Fairness	a. Establish equity objectives for implementation of the selected AI solution.

	measures		b. Identify the most appropriate fairness metrics to use for the selected AI product and its design.
	4. Design AI solution workflow	Fairness, Fitness for purpose, Transparency	a. Ensure that the solution design is informed by meaningful and pragmatic recommendations from members of disadvantaged patient subgroups.<br>b. Ensure that the solution design promotes inclusivity of clinical end-users and usability of the solution.<br>c. Design complementary non-technical solution components required to achieve equity objectives for implementation of the solution.<br>d. Align clinical end-users and organizational leaders to achieve equity objectives.
	5. Generate evidence of safety, efficacy and equity	Accountability, Fairness, Reliability and validity	a. Assess completeness and quality of local data required to construct model features across disadvantaged patient subgroups.<br>b. Seek an approval from an institutional review board, ethical review board, or research ethics board to access and use local healthcare prospective data.<br>c. Analyze label choice bias across disadvantaged and advantaged patient subgroups.<br>d. Ensure that the model features are relevant to its actual label and capture the same meanings across disadvantaged and advantaged patient subgroups.<br>e. Identify potential hidden stratification that masks unequal model performance between disadvantaged and advantaged patient subgroups.<br>f. Assess model performance and compare it between disadvantaged and advantaged patient subgroups.<br>g. Determine which SDOH and demographic data are appropriate to be included in the model to minimize potential risk of worsening health inequities.<br>h. Assess prospective model performance and compare it to retrospective model performance across disadvantaged and advantaged patient subgroups.<br>i. Perform comprehensive assessment of model performance across disadvantaged and advantaged patient subgroups.<br>j. If the model performs worse for a certain patient subgroup on local prospective healthcare data, consider adapting the model or its use to help minimize negative impacts on inequities.<br>k. Ensure that model performance aligns with the equity objectives.<br>l. Conduct a prospective pilot study to validate whether the AI solution achieves equity objectives.
Clinical integration	6. Execute AI	Accountability, Fairness,	a. Document information about the model development and implementation and share it with clinical end-users, members of

	solution rollout	Transparency	disadvantaged patient subgroups, and others who may be affected by use of the model.<br>b. Educate clinical end-users about potential bias in using the solution.<br>c. Where applicable, seek an approval from an institutional review board, ethical review board, or research ethics board to implement and use the AI solution in clinical practice.<br>d. After rollout, continue to seek feedback from clinical end-users and members of disadvantaged and advantaged patient subgroups to achieve equity objectives.
Lifecycle management	7. Monitor the AI solution	Fairness, Reliability and validity, Transparency	a. Regularly monitor the model performance across disadvantaged and advantaged patient subgroups.<br>b. Regularly monitor the work environment of the solution across disadvantaged and advantaged patient subgroups.<br>c. Regularly monitor health inequities across disadvantaged and advantaged patient subgroups.
	8. Update or decommission the AI solution	Accountability, Fairness, Reliability and validity, Transparency	a. Determine updates to the model or its work environment.<br>b. If updating the model or its work environment does not improve model performance or fails to improve progress towards equity objectives, consider decommissioning the model.<br>c. If the model successfully achieved equity objectives and there is interest to expand model use, evaluate appropriate technical and non-technical resources to expand the AI solution to new settings.
