# Supplementary material for: Development and preliminary testing of Health Equity Across the AI Lifecycle (HEAAL): A framework for healthcare delivery organizations to mitigate the risk of AI solutions worsening health inequities
Source: PLOS Digit Health. 2024 May 9;3(5):e0000390. doi: 10.1371/journal.pdig.0000390 (PMC11081364; doi:10.1371/journal.pdig.0000390)
Supplement: Attachment — Submitted filename: Response to Reviewers.docx [file pdig.0000390.s003.docx]

Additional Editor Comments (if provided):

This is a solid and useful piece of work. 
My sole concern is about the choice  (also noted by the reviewer) made by the authors to focus the manuscript on a description of the process that led to the framework rather explaining and commenting the framework itself.
The ms makes too much out of the process that lead to the proposed framework (p. 7-12).
The ms would gain interest if the emphasis was placed on the description of the framework itself. 
In particular, the main information brought by this publicaion is contained in Table 1. It should be part of the ms for clarity and informativeness. 
I am afraid this involves a complete rewriting of the paper but this this in order since in its current form, the text sounds more like the minutes of a meeting rather than like a scientific article.

Thank you for reviewing our manuscript. We truly appreciate your feedback.

We acknowledge that we highlighted the method a lot in our manuscript. We intentionally took this approach because we wanted to be transparent in describing (1) how different types of participants were engaged across different stages of developing the framework, (2) what contributions participants made in each of the stages, and (3) how their contributions were gathered and converged in a systematic manner. Knowing that utilizing a design research method is unconventional in scientific research, we wanted to be transparent about the rigorous methodology followed in our approach. We understand that many other teams develop frameworks among listed co-authors and we want to ensure that we differentiate our contribution with a focus on methodology. We hope that our detailed descriptions is understood as a methodological contribution that helps future researchers utilize participatory design research for their own work. We have now explicitly stated this methodological contribution as a secondary research objective of the paper (lines 19-21 on page 5).

Concurrently, we agree that our manuscript would gain greater interest with more emphasis placed on framework itself. Thus, in our revision, we shorten the description of the process and expand further on the description of the framework. In the results section, we delve deeper into the descriptions of five domains of assessment (pages 11-13), explicitly state reasons for adopting the key decision point framework in the development of HEAAL (lines 17-25 on page 14) and illustrate the overview of HEAAL by demonstrating how it can be used to achieve health equity (pages 17-18).

Minor comments:
Abstract is clear but difference between reliability and validity not clear at this stage.

In the revision, we include the clear definitions of reliability and validity in lines 12-17 on page 13. Inclusion of the definitions should clarify the difference between the two concepts.

p.7 "Design researchers collected responses from framework developers and mapped them
on a Miro board, an online whiteboard with infinite canvas, using sticky notes".
The fact that a Miro board was used is not a crucial information. 
It is fully clear to me what design researchers and frmework deveopers are. Please explain.

In the revision, we’ve removed the description about the Miro board.

Framework developers create a scaffolding of the framework and develop its procedures. Design researchers facilitate the framework design process by collecting and synthesizing data from all other participants. They do not generate the content of the framework themselves. This explanation is added to lines 6-12 on page 6.

p.8 Section "Synthesize key insights of the workshop" is not informative. Please rewrite or remove.

We have re-written this section to promote reproducibility of our methods. This section (lines 6-10 on page 8) now describes how the key insights of the workshop shared by 77 participants were synthesized and incorporated into the content of the framework.

p.8 "Then, design researchers converted the contents on the Miro board
to a single Word document" this information is immaterial.

In the revision, we’ve removed this information.

Figure 3 is not informative and could be removed.

In the revision, we’ve removed this figure.

Table 2 does not seem to be so informative neither to contain any information not contained in Table 5.

In the revision, we’ve removed this table and added a brief description of how procedures for evaluating an existing solution and a new solution may be different in lines 1-10 on page 15.

p.15 I think the sentence "To make a distinction between
two scenarios, procedures for evaluating an existing AI solution are written in red and black.
Procedures for evaluating a new AI solution are written in blue and black (Table 2)" actually refers to Table 5.

Thank you for catching this error. In response to the reviewer’s comment below, Table 5 has transformed into Figure 4 (on page 17) for enhanced visualization.

Data availability statement "Data used in this submission can be accessed in Supporting Information and will be made publicly available on healthaipartnership.org". 
This statement is a bit confusing. What data are you refering to? The Supporting Information document does not seem to contain any "data". Any data intended to be shared should be made available for the review process.

By “data,” we meant “the HEAAL framework.” As we anticipate the HEAAL framework to be continuously improved and adapted over time (lines 23-25 on page 24), we wanted to say that the entire HEAAL framework would be available in the Supporting Information documents and on our HAIP website (healthaipartnership.org).

In the original data availability statement, we were confused with the definition of “data.” As you pointed out, we are not actually sharing data. To correct this, we have updated the data availability statement in the submission portal.

To keep readers informed on the evolving nature of the framework and where they can access updates, we’ve added information about the framework updates and the HAIP website in the conclusion section (line 23 on page 24 – line 2 on page 25).

Please also address carefully the numerous thoughtful comments made by the reviewer.

Please see our responses to the reviewer’s comments in the next pages.

[Note: HTML markup is below. Please do not edit.]

Reviewers' comments:

Reviewer's Responses to Questions

<font color="black"><b>Comments to the Author</b><br><br>
1. Does this manuscript meet PLOS Digital Health’s <a href="[https://urldefense.com/v3/__https://journals.plos.org/digitalhealth/s/journal-information*loc-criteria-for-publication__;Iw!!OToaGQ!oVyn7yTczyS2Z6zUYUB_v9OfS-M9X6L54ViUb7TYxT-qw0hKrf_ODOSa9cca0ZWVBcUD4SFzSTKjVTYEHFQ4nA$](https://urldefense.com/v3/__https:/journals.plos.org/digitalhealth/s/journal-information*loc-criteria-for-publication__;Iw!!OToaGQ!oVyn7yTczyS2Z6zUYUB_v9OfS-M9X6L54ViUb7TYxT-qw0hKrf_ODOSa9cca0ZWVBcUD4SFzSTKjVTYEHFQ4nA$) " target="_blank">publication criteria</a>? Is the manuscript technically sound, and do the data support the conclusions? The manuscript must describe methodologically and ethically rigorous research with conclusions that are appropriately drawn based on the data presented.</font>

Reviewer #1: Yes

-------------------

<font color="black">2. Has the statistical analysis been performed appropriately and rigorously?</font>

Reviewer #1: Yes

--------------------

<font color = "black">3. Have the authors made all data underlying the findings in their manuscript fully available (please refer to the Data Availability Statement at the start of the manuscript PDF file)?<br><br> The <a href="[https://urldefense.com/v3/__https://journals.plos.org/digitalhealth/s/data-availability__;!!OToaGQ!oVyn7yTczyS2Z6zUYUB_v9OfS-M9X6L54ViUb7TYxT-qw0hKrf_ODOSa9cca0ZWVBcUD4SFzSTKjVTY8cRppVA$](https://urldefense.com/v3/__https:/journals.plos.org/digitalhealth/s/data-availability__;!!OToaGQ!oVyn7yTczyS2Z6zUYUB_v9OfS-M9X6L54ViUb7TYxT-qw0hKrf_ODOSa9cca0ZWVBcUD4SFzSTKjVTY8cRppVA$) " target="_blank">PLOS Data policy</a> requires authors to make all data underlying the findings described in their manuscript fully available without restriction, with rare exception. The data should be provided as part of the manuscript or its supporting information, or deposited to a public repository. For example, in addition to summary statistics, the data points behind means, medians and variance measures should be available. If there are restrictions on publicly sharing data—e.g. participant privacy or use of data from a third party—those must be specified.</font>

Reviewer #1: Yes

--------------------

<font color = "black">4. Is the manuscript presented in an intelligible fashion and written in standard English?<br/><br/>PLOS Digital Health does not copyedit accepted manuscripts, so the language in submitted articles must be clear, correct, and unambiguous. Any typographical or grammatical errors should be corrected at revision, so please note any specific errors here.</font>

Reviewer #1: Yes
--------------------

<font color = "black">5. Review Comments to the Author<br/><br/>Please use the space provided to explain your answers to the questions above. You may also include additional comments for the author, including concerns about dual publication, research ethics, or publication ethics. (Please upload your review as an attachment if it exceeds 20,000 characters)</font>

Reviewer #1:

First, I think this is an incredibly important topic, albeit a complex one, and still a gap.

The solutioning process and content give every indication of having come from seasoned professionals with a granular understanding of the AI adoption process, as well as AI creation, although this manuscript reads as through the lens of AI adopters.  

I have almost complete confidence in the quality and comprehensiveness of the output, but my concerns are in the complexity of the communication of said output.

Thank you for reviewing our work and sharing your insights.

When we were building the framework, we had healthcare delivery organization leaders as an intended audience/user of the framework. We hoped that the framework would be able to offer actionable guidance to those who adopt AI in their settings (line 25 on page 4 – line 2 on page 5, lines 16-20 on page 14). For this reason, we recruited various stakeholders with prior experience of AI adoption from healthcare delivery organizations and ecosystem partners as participants of the current project (line 24 on page 5 – line 10 on page 6) and conducted rounds of usability testing (pages 9-10).

We understand that the framework is not simple and may seem intimidating to some users and readers due to its comprehensive nature. In the revision, we noted it as a limitation (lines 7-10 on page 24).

In response to your comment below (which we appreciated very much), we’ve created a figure that shows the overview of the framework (Figure 4, page 17). We hope that the addition of this visual aid enhances the communication.

1) Similar recent paper

First, a quick compare and contrast to another recent paper - Abràmoff, M.D., Tarver, M.E., Loyo-Berrios, N. et al. Considerations for addressing bias in artificial intelligence for health equity. npj Digit. Med. 6, 170 (2023). [https://urldefense.com/v3/__https://doi.org/10.1038/s41746-023-00913-9__;!!OToaGQ!oVyn7yTczyS2Z6zUYUB_v9OfS-M9X6L54ViUb7TYxT-qw0hKrf_ODOSa9cca0ZWVBcUD4SFzSTKjVTaQcpVFsQ$](https://urldefense.com/v3/__https:/doi.org/10.1038/s41746-023-00913-9__;!!OToaGQ!oVyn7yTczyS2Z6zUYUB_v9OfS-M9X6L54ViUb7TYxT-qw0hKrf_ODOSa9cca0ZWVBcUD4SFzSTKjVTaQcpVFsQ$) 

Written more through the lens of an AI practitioner, it mapped easier to existing mental frameworks and most of the text was spent understanding examples of what bias means in health AI and how it is introduced.  Concept, design, develop, validate, access, monitoring as portrayed on a visual graphic.   

Note:  This framework maps more or less to reviewed paper’s buckets, they have chosen to build upon HAIP prior work.  Maybe their framework is more intuitive for the intended audience, I cannot judge.  The other seems simpler from an MLOps perspective.   

With respect to the reviewed paper, I assume it is aimed at the very same and important problem, but is NOT redundant as the intent seems in providing a very clear roadmap/checklist for the holistic set of health system adoption stakeholders to evaluate up front and monitor equity risks and effects.  

Primarily I mention for the editor, nonetheless, would be good for the authors also to consider the other paper and if any cite or alignment needed as appropriate - it's recent, understandably.    Also, suggest to do a quick last check for other recent papers, seems to have become a current topic. 

Thank you for suggesting a recent paper. We’ve added it to our reference (reference number 34). Prior to developing HEAAL, we reviewed recent papers (lines 24-26 on page 4). However, we believed them to be difficult for healthcare delivery organization leaders to use, as there was no comprehensive set of actionable procedures (line 26 on page 4 – line 7 on page 5).

The HEAAL framework was built upon a key decision point framework developed by HAIP. In our current research, we chose to use this key decision point framework because it was generated by a large community of healthcare professionals and ecosystem partners who had experience of AI adoption and knowledge in both AI technology and clinical work environment. The key decision point framework was designed for healthcare delivery organizations, who are our primary audience. As we aim to develop practical guidance to assess health equity through the HEAAL framework, we believed that organizing our research findings around this community-informed framework (vs. other existing frameworks) would be more useful in practice. We’ve incorporated this rationale in lines 16-25 on page 15.

2)  Communication suggestions/simplification

From a pragmatic point of view, the paper is fine.  “Here is the high quality checklist of 37 bullets, here is why you should have confidence in the process”.    But since it seems the authors understand the importance of onboarding, change management, adoption, etc, I would suggest that adding a few easier to digest aspects to the paper might impact a larger audience, beyond those already determined to implement it.

This is a great point. In the revision, we've incorporated your suggestion in the results section lines 20-25 on page 14.

2a) (minor) A minor point first, it does seem a particularly high percentage of text goes to the process, and is ordered first.  I do believe the process is even valuable in its own right.  But is the secondary contribution of the paper, with purpose simply to establish confidence of output.  Just be mindful.

In the revision, we’ve shortened the description of the process and elaborate further on the description of the framework. In the results section, we delve deeper into the descriptions of five domains of assessment (pages 11-13), explicitly state reasons for adopting the key decision point framework in the development of HEAAL (lines 17-25 on page 14) and illustrate the overview of HEAAL by demonstrating how it can be used to achieve health equity (pages 17-18).

2b)  (potentially larger impact but still minor revision)  On output communication, for example, I could strongly suggest a simplified visual, mapping 8 process steps to main risks/checks but at a higher level form.  (“flag training data for bias: representation, label choice, measurement, etc”)  or whatever the authors feel is the most intuitive visual might be.    i.e. less words, less bullets, higher level, more visual.

Again, I make this type of suggestion not to be critical of content, but rather because I feel the addressed problem is important, that the process was well thought out, and hence it would be ideal if there was an easier way for it to be circulated and socialised.

Thank you for the suggestion. In the revision, we’ve replaced the Table 5 (with more words and bullet points) with Figure 4 (less words, no bullet point, more visual) to try to enhance the communication.

2c) (minor) Beyond that, but along the same lines, perhaps more words spent on intuition around the 8 buckets and associated risks/steps.    Is there any more to be added in granularity or description as far as the ethical metrics, etc.   I trust the authors will know better the intended audience and proficiency in topic.  Visually, authors could consider description or nick name in small text to Figure 6 ABCD boxes.

A new section named “achieving health equity” has been added to address this comment (pages 17-18). This section outlines the procedures of the framework across eight key decision points. It offers descriptions of key procedures at each decision point and illustrates how an organization can advance towards equity objectives. It describes the process of defining baseline inequities, setting equity objectives, and evaluating whether the AI solution achieves the equity objectives.

Description has been added within the ABCD boxes in Table 4 (line 10 on page 19).

3)  (food for thought). Lastly, I am pasting in more of a “Health equity for non-experts” read up I have seen recently in smw as I reside in Switzerland, strictly as an example of simple communication.   However it will not map 1:1 for the authors purposes but may offer food for thought. 

The pursuit of health equity in the era of artificial intelligence
[https://urldefense.com/v3/__https://smw.ch/index.php/smw/article/view/3286/5530__;!!OToaGQ!oVyn7yTczyS2Z6zUYUB_v9OfS-M9X6L54ViUb7TYxT-qw0hKrf_ODOSa9cca0ZWVBcUD4SFzSTKjVTby4Ce0mQ$](https://urldefense.com/v3/__https:/smw.ch/index.php/smw/article/view/3286/5530__;!!OToaGQ!oVyn7yTczyS2Z6zUYUB_v9OfS-M9X6L54ViUb7TYxT-qw0hKrf_ODOSa9cca0ZWVBcUD4SFzSTKjVTby4Ce0mQ$) 
[https://urldefense.com/v3/__https://doi.org/10.57187/smw.2023.40062__;!!OToaGQ!oVyn7yTczyS2Z6zUYUB_v9OfS-M9X6L54ViUb7TYxT-qw0hKrf_ODOSa9cca0ZWVBcUD4SFzSTKjVTZwIaLZiQ$](https://urldefense.com/v3/__https:/doi.org/10.57187/smw.2023.40062__;!!OToaGQ!oVyn7yTczyS2Z6zUYUB_v9OfS-M9X6L54ViUb7TYxT-qw0hKrf_ODOSa9cca0ZWVBcUD4SFzSTKjVTZwIaLZiQ$) 

Thank you for the suggestion. We hope that our communication has become clearer in the revision. However, we are aware that the HEAAL framework is not simple. We state this as a limitation in the “limitations and future directions” section (lines 7-10 on page 24).

Additional thoughts:

This is touched upon at various points, but an algorithm which leads to an increased inequity in one market could have a significant outcomes boost in another markets, there are different bars for risk-return.  The specific examples that come to mind are mostly out of US.

Yes, that’s right. The response to this comment is reflected in the first paragraph of the “implications for practice” section on page 22. To convey our argument more clearly, we added a sentence acknowledging that different contexts could reach different conclusions about the same algorithm (lines 22-25 on page 22)

What does the product mindset for this look like?

We focus on all stages of the AI product lifecycle with a focus on the perspective of healthcare delivery organizations. In response to evolving nature of AI technologies and landscape of health disparities, we plan to continue refining, improving, and adapting the HEAAL framework. Please see the “conclusion” section on pages 24-25.

Also it may be instructive to remind the reader that for some tasks, humans are also just predictive models that have their own biases.   Although I believe this is captured inherently in the framework.

HEAAL contains procedures regarding educating users about potential bias that they may have and its consequences in the sixth decision point. In the “achieving health equity” section, the role of communication and education is now described (lines 18-22 on page 18).

--------------------

<font color = "black">6. PLOS authors have the option to publish the peer review history of their article (<a href="[https://urldefense.com/v3/__https://journals.plos.org/digitalhealth/s/editorial-and-peer-review-process*loc-peer-review-history__;Iw!!OToaGQ!oVyn7yTczyS2Z6zUYUB_v9OfS-M9X6L54ViUb7TYxT-qw0hKrf_ODOSa9cca0ZWVBcUD4SFzSTKjVTZ6JPZw6A$](https://urldefense.com/v3/__https:/journals.plos.org/digitalhealth/s/editorial-and-peer-review-process*loc-peer-review-history__;Iw!!OToaGQ!oVyn7yTczyS2Z6zUYUB_v9OfS-M9X6L54ViUb7TYxT-qw0hKrf_ODOSa9cca0ZWVBcUD4SFzSTKjVTZ6JPZw6A$) " target="_blank">what does this mean?</a>). If published, this will include your full peer review and any attached files.<br><br>

<b>Do you want your identity to be public for this peer review?</b> If you choose “no”, your identity will remain anonymous but your review may still be made public.<br><br>

For information about this choice, including consent withdrawal, please see our <a href="[https://urldefense.com/v3/__https://www.plos.org/privacy-policy__;!!OToaGQ!oVyn7yTczyS2Z6zUYUB_v9OfS-M9X6L54ViUb7TYxT-qw0hKrf_ODOSa9cca0ZWVBcUD4SFzSTKjVTapotUntA$](https://urldefense.com/v3/__https:/www.plos.org/privacy-policy__;!!OToaGQ!oVyn7yTczyS2Z6zUYUB_v9OfS-M9X6L54ViUb7TYxT-qw0hKrf_ODOSa9cca0ZWVBcUD4SFzSTKjVTapotUntA$) " target="_blank">Privacy Policy</a>.</font>

Reviewer #1: Yes: Nicholas W. Kelley
--------------------

[NOTE: If reviewer comments were submitted as an attachment file, they will be attached to this email and accessible via the submission site. Please log into your account, locate the manuscript record, and check for the action link "View Attachments". If this link does not appear, there are no attachment files.]

While revising your submission, please upload your figure files to the Preflight Analysis and Conversion Engine (PACE) digital diagnostic tool, [https://urldefense.com/v3/__https://pacev2.apexcovantage.com/__;!!OToaGQ!oVyn7yTczyS2Z6zUYUB_v9OfS-M9X6L54ViUb7TYxT-qw0hKrf_ODOSa9cca0ZWVBcUD4SFzSTKjVTbKaIzv5Q$](https://urldefense.com/v3/__https:/pacev2.apexcovantage.com/__;!!OToaGQ!oVyn7yTczyS2Z6zUYUB_v9OfS-M9X6L54ViUb7TYxT-qw0hKrf_ODOSa9cca0ZWVBcUD4SFzSTKjVTbKaIzv5Q$) . PACE helps ensure that figures meet PLOS requirements. To use PACE, you must first register as a user. Registration is free. Then, login and navigate to the UPLOAD tab, where you will find detailed instructions on how to use the tool. If you encounter any issues or have any questions when using PACE, please email PLOS at [figures@plos.org](mailto:figures@plos.org). Please note that Supporting Information files do not need this step.

All figures used in the manuscript are uploaded to PACE. Following the submission rule, all figures are removed from the manuscript and provided separately.
